# Investigation of relationship between occupational exposure to aerosol and sleep problems: A systematic review and meta-analysis

**DOI:** 10.1371/journal.pone.0321515

**Published:** 2025-05-09

**Authors:** Amir Hossein Khoshakhlagh, Saeid Yazdanirad, Agnieszka Gruszecka-Kosowska, Christopher L. Drake, Wickwire Emerson

**Affiliations:** 1 Department of Occupational Health, School of Health, Kashan University of Medical Sciences, Kashan, Iran; 2 Department of Occupational Health, School of Health, Shahrekord University of Medical Sciences, Shahrekord, Iran; 3 Social Determinants of Health Research Center, Shahrekord University of Medical Sciences, Shahrekord, Iran; 4 Department of Environmental Protection, Faculty of Geology, Geophysics, and Environmental Protection, AGH University of Krakow, Al. Mickiewicza 30, 30-059, Krakow, Poland; 5 Henry Ford Health System, Sleep Disorders and Research Center, Detroit, Michigan, United States of America; 6 Psychiatry and Medicine, Section Head, Sleep Medicine, University of Maryland School of Medicine, Baltimore, United States of America; Satyawati College, University of Delhi, INDIA

## Abstract

There are various occupational and industrial activities that are associated with the production of suspended particles. Little is known about sleep disturbance caused by exposure to aerosol exposure. Presented systematic review and meta-analysis study aimed to investigate the impact of various aerosols during occupational exposure on sleep. A systematic search in Scopus, Web of Science, PubMed, Embase, and Medline databases was performed until 20 February 2024. Three sets of keywords and their possible combinations were used in the search algorithm. To evaluate the quality and risk of bias in studies, the Joanna Briggs Institute (JBI) tools and risk of bias in non-randomized studies of exposure (ROBINS-E) instruments were applied, respectively. The pooled values were also computed by meta-analysis. Based on inclusion/exclusion criteria, 23 articles were entered into the review. 9 out of 11 articles with high quality (81.82 percent), 8 out of 9 articles with moderate quality (88.89 percent), and 2 out of 3 articles with low quality (66.67 percent) indicated that aerosol exposure had a meaningful negative effect on sleep among workers in various occupations. Among articles, 69.6% (N = 16) were given a high risk of bias rating, 13.0% (N = 3) were rated as moderate risk of bias, and 17.4% (N = 4) were rated as low risk of bias. The results of the meta-analysis indicated that the pooled value of the prevalence in the cross-sectional, cohort, and case control studies was 42.35 (95%CI [34.55, 50.16]), 10.82 (95%CI [6.76, 14.87]), and 35.70 (95%CI [13.96, 57.45]), respectively. Also, the results of the meta-analysis showed that the pooled values of the odds ratio in the cross-sectional and cohort studies were 1.82 (95% CI [1.43, 2.21]) and 1.73 (95% CI [1.49, 1.96]), respectively. Totally, most studies indicated that various sources of occupational aerosol exposure significantly affected sleep among employees.

## Introduction

The definition states that air aerosols are fine solid particles and liquid droplets that are suspended in the air. They can occur in ambient atmosphere and in occupational environments [1, 2]. Aerosols or suspended particles form an important group of air pollutants in work environments [2]. Occupational aerosols contain a wide range of particles’ diameters from nanoparticles with the diameter of nanometers scale to coarse particles with a range of diameters from micrometers and even macroscopic particles at the millimeter scale [3]. On average the size of aerosol particles is in the range from 0.002 to 100 μm [3]. The four most common classifications of aerosols in the occupational environment include dust, fume, mist, and smoke. Dust is the fraction of aerosols containing fine particles of solid matter [4]. Fumes are ultrafine and inhalable solid particles that are generated as the effect of gas condensation as the consequence of the sublimation of molten materials [5]. Mist is defined as tiny water droplets suspended in the air [6]. Smoke is a suspension of airborne particulates emitted when a material undergoes combustion or pyrolysis [7]. It is important to know that liquid droplets can be considered particles that may act like solid particles in the atmosphere [8].

There are various occupational and industrial activities that are associated with the production of suspended particles. For example, working in hard rocks and coal mines where silica and coal dust are created [9], working in environments with processes that are accompanied by high temperatures, such as electric arc welding, flame cutting, casting, and metal melting, in which fine metal oxides are created [10], working in occupations that mist is produced, such as turning and painting [11], and working in environments where materials are transformed into particulates such as cleaning and sawing [12]. Aerosols can react with or be absorbed by body tissues and lead to adverse health effects. These adverse effects may vary from simple irritation to a systemic disease, such as asthma, bronchitis, heart diseases, and cancers, depending on the size, shape, density, concentration, chemical properties of the aerosol, duration of contact, and other factors [1].

Little is known about sleep disturbance caused by exposure to aerosol exposure. The definition states that the quality and amount of sleep disorders are called sleep disturbances. Clinically severe and chronic sleep disturbances can result in distress and fatigue during the day as well as functional impairment, resulting in insomnia disorder [13]. Exposure to aerosols can cause sleep disturbance through various pathways. Exposure to some chemical aerosols can impact the nervous system directly or indirectly and can be associated with adverse neurobehavioral effects, including insomnia [14]. Moreover, the psychological effects due to aerosol exposure, including annoyance, health concerns, and anxiety because of direct exposure to dangerous aerosols may also lead to sleep disturbances [15]. Also, aerosol exposure can result in chronic illnesses, starting from throat dryness, through chest tightening, coughing, wheezing, and difficulties during breathing up to chronic bronchitis, which may exacerbate sleep disorders [16]. Furthermore, the exposure to some aerosols may impact on serotonin secretion. Serotonin is an important neurotransmitter involved in sleep-wake regulation. Low levels of this hormone are linked with sleep disturbances and functional impairment including excessive sleepiness [17].

Some review research investigated correlations between exposure to environmental air pollutants and sleep disorders. Boor et al. (2017) in their literature review on the exposure to indoor air pollutants in sleep environments concluded the negative impact on human sleep [18]. The results of the systematic review on air pollution and related negative effects on sleep quality indicated that exposure to air pollutants was the major cause of breathing disorders during sleep among adults [19]. Liu et al. (2021) in a review study on the association between environmental pollution exposure and sleep outcomes revealed that air pollution is responsible for various sleep problems [20].

Also, occupational exposure to various air pollutants could affect the quality of the sleep during night. Occupational exposure is different from environmental exposure in terms of types of pollutants, their concentrations, exposure duration, and time of exposure. Thus, health effects related to sleep during occupational exposure to aerosols may differ from those arising from environmental exposure. On the other hand, the effects of exposure to different aerosols, including dust, mist, fume, and smoke, on sleep problems may be different. In order to investigate the health effects of exposure to various aerosols in working environments on sleep quality, a systematic review and meta-analysis study was performed. Also, it was aimed to represent the prevalence of sleep problems due to exposure to aerosols in the reviewed study.

## Materials and methods

The systematic review and meta-analysis carried out in our study was done based on the guidelines on the Preferred Reporting Items for Systematic Reviews and Meta-Analyses (PRISMA) guidelines [21] and was registered in the International Prospective Register of Systematic Reviews (PROSPERO) (reference number: CRD42023391106). Furthermore, the PECO framework, for population, exposure, comparator, and outcome, has been provided to represent a clearer depiction of the relationship between occupational exposure to aerosols and sleep problems [22]. The details of this framework are summarized in Table 1. In this systematic review and meta-analysis study, the PECO framework informed the formulation of the research question, as well as the search strategy and criteria for inclusion and exclusion.

**Table 1:** PECO (population, exposure, comparator, and outcome) description.

PECO	Evidence
Population	All working population
Exposure	Occupational exposure to aerosols
Comparator	Variability in sleep quality due to exposure to aerosols
Outcome	Sleep problems

### Literature search

It was aimed to involve articles with proper quality in English from valid databases. Therefore, the literature search was performed without time limitation until 20 February 2024 in Scopus, Web of Science, PubMed, Embase, and Medline databases. Three sets of keywords and their possible combinations were used in the search algorithm. In the first set of keywords “sleep OR circadian OR insomnia OR dyssomnia OR parasomnia OR hypersomnia OR somnolence OR sleepiness” were used. In the second group, the following keywords were used: “fiber OR fibre OR dust OR soot OR smog OR fly ash OR fog OR nanoparticle OR aerosol OR biological agent OR fume OR smoke OR spray OR mist” and in the third group, the following keywords were used: “industry OR industrial OR occupation OR occupational OR workplace OR employee OR workforce OR worker”. The search strategy of keywords was as follows.

((((((((((((((((((((Sleep[Title/Abstract]) OR (sleepiness[Title/Abstract])) OR (circadian[Title/Abstract])) OR (dyssomnia[Title/Abstract])) OR (parasomnia[Title/Abstract])) OR (Insomnia[Title/Abstract])) OR (hypersomnia[Title/Abstract])) OR (“restless legs syndrome”[Title/Abstract])) OR (“RLS”[Title/Abstract])) OR (“Willis Ekbom Disease”[Title/Abstract])) OR (“periodic limb movement disorder”[Title/Abstract])) OR (narcolep[Title/Abstract])) OR (paroxysmal[Title/Abstract])) OR (“gelineau Syndrome”[Title/Abstract])) OR (somnolence[Title/Abstract])) OR (“nocturnal myoclonus syndrome”[Title/Abstract])) OR (nightmare*[Title/Abstract]) AND (((((((((((((((Fiber*[Title/Abstract]) OR (fibre*[Title/Abstract])) OR (soot[Title/Abstract])) OR (smog[Title/Abstract])) OR (“fly ash”[Title/Abstract])) OR (fog[Title/Abstract])) OR (Nanoparticle*[Title/Abstract])) OR (fume*[Title/Abstract])) OR (smoke[Title/Abstract])) OR (“Biological agent*”[Title/Abstract])) OR (mist[Title/Abstract])) OR (spray*[Title/Abstract])) OR (dust[Title/Abstract])) OR (aerosol*[Title/Abstract]) AND ((((((((Occupation*[Title/Abstract]) OR (Industry[Title/Abstract])) OR (Industrial[Title/Abstract])) OR (workplace*[Title/Abstract])) OR (Employee*[Title/Abstract])) OR (Workforce*[Title/Abstract])) OR (worker*[Title/Abstract]).

### Eligibility criteria

As inclusion criteria, all types of sleep problems were considered in this study as the purpose of this study was to investigate the effects of occupational exposure to aerosols on sleep. Also, the studies performed on the working population were selected to enter into the review. Studies included in the review were retrospective, cohort, cross-sectional, case-control, and experimental research written in English without time restrictions. Given that most high-quality studies are published in English in valid databases, it was aimed to select these studies. In addition, given the wide range of non-English languages and the lack of proficiency of the research team in these languages for data extraction, only studies written in English were included in the review. As exclusion criteria, the studies were excluded in our paper including meta-analysis and review studies, case reports, editorial letters, conference papers, and trial studies. No filters were used to restrict publication year, gender, age, and population type. Animal studies were also not taken into consideration in this paper.

### Study selection

All papers obtained from investigated databases were transferred to the Endnote. After removing duplicates, two reviewers (A.H.KH. and S.Y) independently analyzed titles and abstracts to select eligible studies. After that, irrelevant and those studies that did not have inclusion criteria described were removed. After this step, two independent reviewers (A.H.KH. and S.Y) read full texts of the remained research to confirm inclusion/exclusion criteria.

### Data extraction

After designating the appropriate for this systematic review and meta-analysis paper, the following information was elucidated by two researchers independently: name of the first author, year of publication, country of research, study type, sample size, job type, gender, age, work experience, pollutant sources, pollutant types, pollutant concentrations, duration of pollutant exposure, information on sleep problem, prevalence of sleep problems, research tool, and obtained outcomes. The outcome is the findings of the studies on the relationship between exposure to aerosol and sleep problems.

### Studies’ quality and bias risk assessment

To evaluate the quality and bias risk of experimental, cohort, case-control, and cross-sectional studies, criteria of appraisal tools by the Joanna Briggs Institute (JBI) were used [23]. The JBI criteria investigate cohort studies that are based on the following criteria: similarity between exposed and non-exposed groups, exposure validity and similarity in both investigated groups, identification and adjustment of confounding factors, validity and reliability of outcomes measurement, sufficiency and implementation of follow-up time, and statistical analysis appropriateness. Additional criteria were used to evaluate experimental studies, namely cause–effect clearness, similar comparisons among participants, among exposures, intervention of participants, control group existence, outcome multiple measurements, follow-up status, comparable outcome measurements, outcome measurement reliability, and statistical analysis appropriateness. Assessment of case-control studies was performed qualitatively using several criteria, that included: outcomes presence or absence in case and control groups, cases and controls matching, similarity criteria for case and control diagnosis, exposure validity and reliability and outcome measurement, exposure period, and statistical analysis appropriateness. Evaluation of the cross-sectional studies was performed using the following criteria: details of criteria inclusion as well as the studied subjects and settings, validity and reliability related to exposure measurement, regular criteria applied for condition measurement, confounding factor recognition, settled strategies of managing the confounding factors, validity and reliability of outcome measurement, and statistical analysis appropriateness. The number of positive responses obtained from the above-described checklists was summed and the categorization of research papers was performed, namely low-quality, moderate-quality, and high-quality articles. Moreover, the potential for bias within the chosen studies was assessed using the risk of bas in Non-randomized Studies-of exposure (ROBINS-E) instrument by two independent evaluators, S.Y. and A.H.Kh. This process involved an examination of seven distinct factors: confounding variables, selection bias, deviations from intended exposures, accuracy of exposure measurement, outcome measurement, missing data, and the clarity of reported findings. Each study underwent a separate ROBINS-E analysis, with outcomes categorized as “low,” “moderate,” or “high” in terms of risk of bias. An overall classification of “high” risk of bias was assigned if any single criterion received a “high” rating. Conversely, a study was deemed to have a “low” risk of bias if all criteria were marked as “low” risk. In cases where these conditions were not met, the study was designated as having a “moderate” overall risk of bias [24].

### Data analysis

Cohen’s kappa index was used to assess the between-raters agreement using the SPSS software [25]. Cohen’s kappa coefficient during the first step of the articles’ selection was equal to 0.91 and during the second step of the articles’ information selection was equal to 0.94 indicating a good agreement rate between the two reviewers. In this study, the pooled values of odds ratios and prevalence related to sleep problems due to exposure to aerosols were computed by meta-analysis. To evaluate heterogeneity in occupational exposure to aerosols, the Cochrane Q-test was utilized [26]. Random effects models were used for meat-analysis in the present study [26]. Additionally, I^2^ was computed as the proportion of variability due to heterogeneity [27]. The I^2^ statistics were employed to measure the extent of variability in a meta-analysis, signifying the proportion of total variance due to heterogeneity [28]. I^2^ values lower than 25 percent show low heterogeneity, values between 25 and 75 percent present moderate heterogeneity, and values higher than 75 percent indicate high heterogeneity [28]. For subgroup analyses, nations were classified based on economic status from low and middle income (LMIC) to high income (HIC) following World Bank classifications [29]. Furthermore, the research was organized into four global regions: Europe, East/southeast Asia/Oceania, the Middle East, North America, and Africa. Based on temporal data, studies were segregated into those conducted in or before 2010 and after 2010. Also, based on the type of aerosol, the studies were categorized as research on dust, mist, fume, and smoke. Statistical analyses were executed with STATA version 14.2.

## Results

### Search strategy and selection of studies

**Fig 1:**
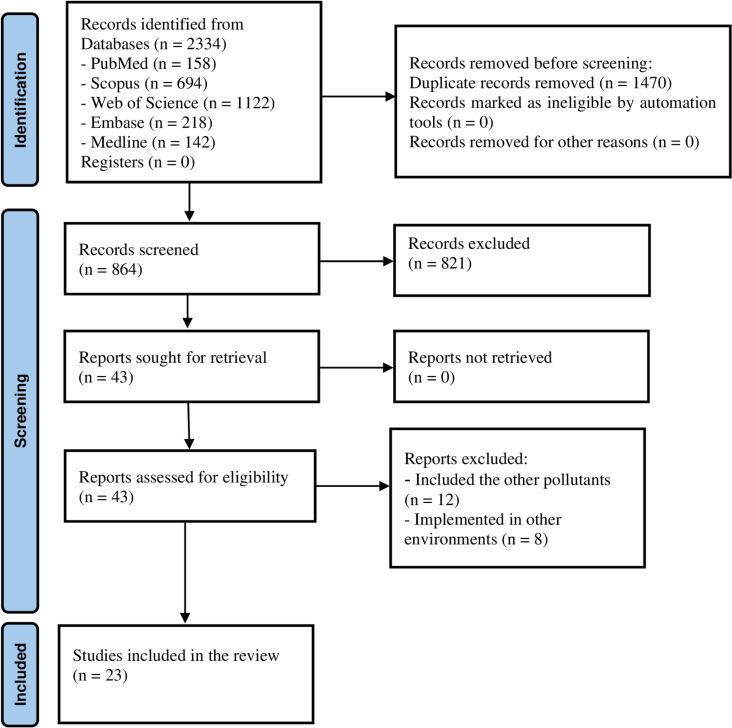
The flow diagram of PRISMA 2020 (preferred reporting items for systematic reviews and meta-analyses).

In the presented systematic review and meta-analysis, in total of 2334 articles were obtained from Scopus, Web of Science, PubMed, Embase, and Medline databases without time restrictions until 20 February 2024. Among this number, 1470 duplicates were found and removed. Titles and abstracts of the remaining 864 articles were screened independently by two researchers. Subsequently, 841 additional articles were excluded due to meeting exclusion criteria or due to lacking inclusion criteria. 23 articles were passed positively of the search and study selection process [30], which they were included in the review. The flow diagram of PRISMA 2020 is presented in Fig 1.

### Specification of the articles

**Fig 2:**
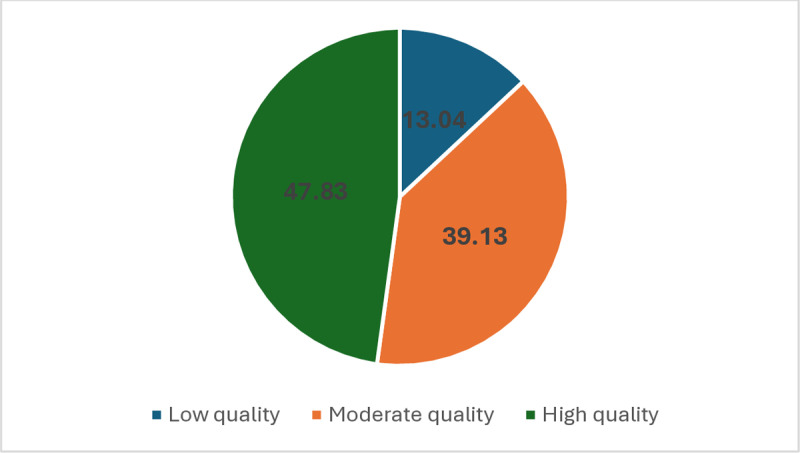
Evaluation of the quality of the reviewed studies.

Among 23 articles that were included in the systematic review and meta-analysis as eligible and related to the topic (Table 2), 11 studies were identified as cross-sectional, eight studies as case-control, one study as experimental, and three were cohort studies. Regarding the country of origin, four studies were from the United States, three studies were from South Korea, two studies each from Iran, India, and China, and one study each from Australia, Belgium, South Africa, Denmark, Taiwan, Turkey, Poland, Nigeria, Uganda, and Spain. Among these studies, three studies were performed on the general working population, one study on workers in a brick factory, one study on military personnel, one study on university students, one study on cosmetologists, one study on painters, three studies on welders, one study on workers in a textile plant, one study on pesticide applicators, six studies on farmers, one study on rescue and recovery workers, one study on workers in the battery industry, one study on soldering workers, and one study on workers in a copper smelting factory. From investigated studies, 11 concerned males, two studies concerned females, and 10 studies concerned both male and female participants. Three studies investigated the effects of smoking on sleep, eight studies surveyed the effect of dust on sleep, nine studies evaluated the effect of mist on sleep, and nine studies assessed the impact of fumes on sleep. Among these articles, six studies investigated sleep disturbance, ten assessed sleepiness, five insomnia, three poor sleep quality, and one obstructive sleep apnea. All studies, except a study, used the subjective tools including questionnaires and scales for measurement of sleep problems. After performing a quality assessment, eleven articles were classified as of high quality (47.83 percent), nine studies as of moderate quality (39.13 percent), and three studies as low-quality studies (13.04 percent). These results are reported in Fig 2.

**Table 2:** The specification of the published research under investigation.

First author (Year)	Country	Sample size	Study Type	Job Type	Range/ mean age (years) and gender	Work experience (year)	Pollutant sources	Concentration	Type of pollutant	Duration of exposure (hour)	Information on sleep (prevalence)	Sleep quality tool	Outcome	Quality
Bowler et al. (2006) [31]	United States	43	Cross-sectional	Bridge welders	43.8 Male	14.20	Welding	0.21 (mg/m^3^, air) and 9.6 (mg/l, blood)	Fume (manganese)	–	Sleep disturbance (79.1%)	Self-reported questionnaire	The exposure to manganese fume showed no significant association with sleep disturbance.	Q2
Chuang et al. (2017) [17]	Taiwan	150	Case-control	Shipyard welders	46.2 Male and female	–	Welding	2.17 (mg/m^3^, air)	Metal fume (Cd, Co, Cu, Mn, Ni, and Pb)	–	Poor sleep quality	Fitbit charges heart rate	Welding workers were longer awake compared to office workers (P < 0.05).	Q1
Ekinci et al. (2014) [32]	Turkey	46	Cross-sectional	Workers in the battery industry	23.51 Male	<5 to>5	–	0.48 (mg/l, blood)	Fume (mercury)	–	Insomnia (67.7%)	Self-reported questionnaire	Insomnia was the most common systemic symptom among the exposed workers.	Q2
Fuhrimann et al. (2023) [33]	Uganda	253	Cross-sectional	smallholder farmers	≤ 39- ≥ 50 Male and female	–	Handling, mixing, and spraying pesticides	0.20 (Glyphosate) 6.54 (Mancozeb) μg/g creatinine	Mist (pesticide)	8	Sleep problems	MOS-SS sleep problem indices	It was observed positive associations between any pesticide application in the last 7 days with all three MOS-SS indices.	Q1
Gallicchio et al. (2011) [34]	United States	961	Cross-sectional	Cosmetologist	42.95 Female	–	Cosmetology activities	–	Mist (cleaning supplies)	–	Sleep disturbance (33.1%)	Sleep survey	Administration of cleaning products indicated a significant association with the reporting of frequent sleep disturbances (OR = 1.64).	Q1
Jamal et al. (2016) [35]	India	374	Case-control	Farmers	37.45 Male	9.88	Handling, mixing, and spraying pesticides	–	Mist (Organophosphate)	–	Insomnia (Overall: 4.0%, control: 3.7%, and sprayers: 4.3%)	General health questionnaire	The rates of insomnia were stated significantly higher among pesticide sprayers than among controls (P = 0.03).	Q2
Jay et al. (2017) [36]	Australia	2961	Cross-sectional	General working population	20 to ≥ 55 Male and female	–	–	–	Dust, smoke, and fume	≥ 8	Sleepiness (People with standard hours: 5.6% and With non-standard hours: 9.6%)	Epworth Sleepiness Scale (ESS)	The workers with non-standard hours of working were more exposed to hazardous factors in workplaces and reported inadequate sleep and excessive sleepiness.	Q2
Lee et al. (2020) [37]	South Korea	78,512	Cohort	General working population	<40 to ≥ 60 Male and female	–	–	–	Dust	–	Insomnia (4.8%) and sleep disturbance (5.9%)	Self-reported questionnaire	Severe exposure levels caused an increase in sleep disturbance or insomnia risk (OR = 1.52).	Q1
Li et al. (2019) [38]	China	1368	Cross-sectional	Greenhouse farmers	46.7 Male and female	–	Handling, mixing, and spraying pesticides	–	Mist (pesticide)	–	Poor sleep quality (1.47%)	Sleep questionnaire	Insufficient protective means in pesticide use was stated as the cause of decreased sleep quality and increased prevalence of nightmares.	Q2
London et al. (1998) [39]	South Africa	247	Case-control	Pesticide applicators	36.9 Male	–	Handling, mixing, and spraying pesticides	–	Mist (Organophosphate)	–	Sleepiness (overall: 36%, people exposed: 43.00%, and non-exposed: 23.00%)	Sleep questionnaire	Current applicators in comparison with non-applicators reported significantly more sleepiness.	Q1
Mohammadyan et al. (2019) [40]	Iran	40	Cross-sectional	soldering workers	35.4 Female	7.8	–	0.09 (mg/m^3^, air) and 0.11 (mg/l, blood)	Fume (lead)	8	Poor sleep quality (67.5%)	Pittsburgh Sleep Quality Index	A significant correlation was stated among sleep quality, blood lead level (P = 0.03, and air lead content (P = 0.02)).	Q1
Mokarami et al. (2020) [41]	Iran	90	Cross-sectional	Workers in a brick factory	35.60 Male	> 1	–	2.8 (mg/m^3^)	Respirable dust	8–12	Sleepiness (13.3%)	Epworth sleepiness scale (ESS) and Stop‑Bang questionnaire	Inhalation dust was able to predict an ESS score showing faulty sleep conditions (OR = 5.35).	Q1
Powell et al. (2020) [42]	United States	100	Cohort	Military personnel	39.75 Male and female	–	Burn pit	–	Smoke	–	Sleepiness (people without exposure:71.0% and with burn pit exposure:69.0%)	Epworth sleepiness scale (ESS) and Stop‑Bang questionnaire	Obstructive sleep apnea (OSA) occurrence was high in both groups but it has no significant difference (P = 0.83).	Q1
Pan et al. (2000) [43]	Denmark	10	Experimental	University students	27.5 Male and female	–	Dust exposure generator	0.394 (mg/m^3^)	Airborne Office Dust	3	Sleepiness	Sleep questionnaire	Exposure to office dust related to industry is responsible for sensory symptoms in the eyes, and nose sleepiness, and physiological changes.	Q2
Qin et al. (2014) [44]	China	606	Case-control	Welders	33.58 Male	–	Welding	–	Fume	–	Insomnia (overall: 40.1%, welders: 42.90%, and non-dust workers: 37.4%)	Sleep questionnaire	Insomnia had no significant difference between welders and non-dust workers (P = 0.126).	Q1
SikCho et al. (2022) [45]	South Korea	36996	Cohort	General working population	15 to ≥ 60 Male and female	–	–	–	Smoke, fume, dust or powder	–	Sleep disturbance (6.3%)	Minimal Insomnia Symptom Scale (MISS)	Exposure to smoke, fumes, and dust was positively associated with sleep disturbance (OR = 2.12).	Q1
Sosan et al. (2009) [46]	Nigeria	150	Cross-sectional	Farmers	20 to ≥ 60 Male	–	Handling, mixing, and spraying insecticide	–	Mist (insecticide)	<5–12	Sleeplessness (40%)	Self-reported questionnaire	Sleeplessness symptoms among farmers suggestive of chronic pesticide poisoning.	Q3
Sohn et al. (2001) [47]	South Korea	8,420	Cross-sectional	Farmers	20–70 Male and female	–	Handling, mixing, and spraying pesticides	–	Mist (pesticide)	6.8 (days per year)	sleeplessness	Sleep survey	Pesticide poisoning was significantly related to sleeplessness (P < 0.01).	Q2
Sunderram et al. (2019) [48]	United States	601	Cross-sectional	Rescue and recovery workers	52.8 Male and female	–	World Trade Center (WTC) disaster	–	Toxic dust and fumes	–	Obstructive sleep apnea (OSA) (75.0%) and Insomnia (52.5%)	Sleep questionnaire, Epworth sleep scale, and Apnea risk evaluation system	New and worsening chronic rhinosinusitis (CRS) compared with no CRS was indicated as a significant risk factor of OSA (OR = 1.80).	Q2
Tripathi et al. (1989) [49]	India	95	Case-control	Painters	32.75 Male	10.63	High-pressure spray painting	0.59 to 4.63 (mg/m^3^, air)	Mist (solvents)	4	Sleepiness	Visual analogue scales	Significant deleterious effects were noted on sleepiness among painters.	Q3
Takam et al. (1988) [50]	Belgium	193	Case-control	Workers in a textile plant	33.5 Male	10.60	Weaving mill	6.40 (mg/m^3^, air)	Dust (cotton)		Daytime sleepiness (overall: 31%)- Restless sleep (overall: 28.3%)	Sleep questionnaire	No significant differences among exposed and control subjects on sleep-related disturbances.	Q3
Walczak et al. (2010) [51]	Poland	37	Case-control	Workers in copper smelting factory	44 Male	18.1	–	0.01 (mg/m^3^, air)	Fume (arsenic)	8	Sleeplessness	Self-reported questionnaire	Sleeplessness was stated as the main functional disorder of the nervous system due to chronic occupational exposure to arsenic.	Q2
Zheng (2023) [52]	Spain	380	Case-control	farmers	39.27 male	12.61	Handling, mixing, and spraying pesticides	–	Mist (Pesticides)	8	Sleep problem (overall: 69.5%, agricultural occupation: 83.1%, and non-agricultural occupation: 56.0%)	Oviedo Sleep Questionnaire	Agricultural employees were found to be at a significantly higher risk of insomnia, especially among those who did not wear protective gloves.	Q1

### Key outcomes

**Fig 3:**
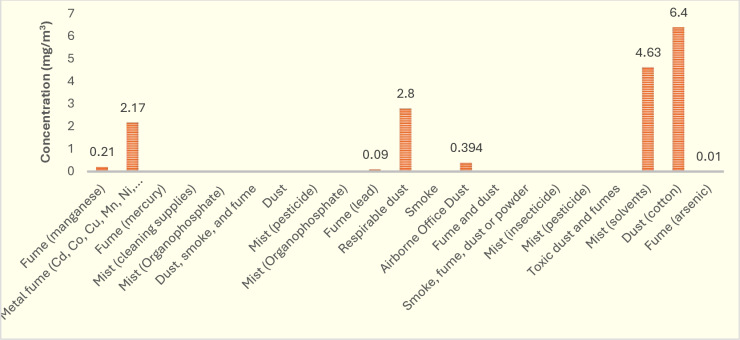
The concentration (mg/m^3^) of aerosols reported in the reviewed study.

**Fig 4:**
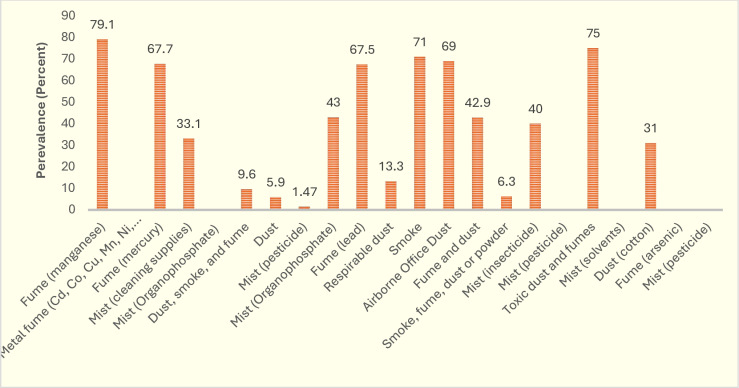
The prevalence of sleep problems due to exposure to aerosols in the reviewed study.

**Fig 5:**
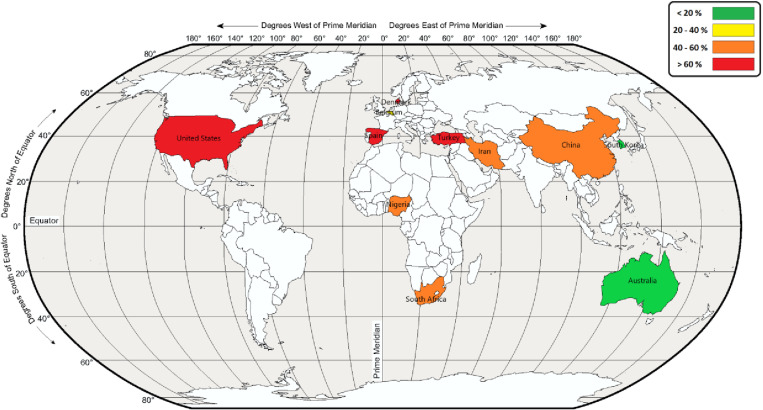
Global map of the prevalence of sleep problems due to occupational exposure to aerosols.

Fig 3 indicates the concentration (mg/m^3^) of aerosols in the reviewed study. Some studies have not reported this concentration. The highest occupational exposure concentrations to aerosols were related to cotton dust (6.40 mg/m^3^) and solvent mist (4.63 mg/m^3^), respectively. Fig 4 also illustrates the prevalence of sleep problems due to exposure to aerosols in the reviewed study. Fig 5 depicts the global map of the prevalence of sleep problems due to occupational exposure to aerosols. Based on this map, the highest sleep problems due to this exposure were observed in Spain due to exposure to pesticide mist (83.1 percent), United States due to exposure to manganese fume (79.1 percent), and United States due to exposure to toxic dust and fumes (75.0 percent), respectively.

Among 11 articles of high quality, nine studies (81.82 percent) showed that the exposure to aerosols among workers in different occupational exposures indicated a significant negative impact on sleep. Two studies analyzed the impact of exposure to smoking on sleep quality, two studies examined the effect of mist exposure on sleep, four studies investigated the effect of fume exposure on sleep, and four studies researched the effect of dust on sleep. The observed negative effects in these studies were sleep disturbances, sleepiness, insomnia, and poor sleep quality. SikCho et al. (2022) pointed out that exposure to smoke, fumes, and dust had a positive association with sleep disturbances among the general working population [OR = 2.12 (95%CI: 1.71–2.63)] [45]. Mokarami et al. (2020) observed that respirable dust was predictive of ESS score (i.e., sleepiness) indicating abnormal sleep status among workers in a brick factory [OR = 5.35 (95%CI: 1.40–19.90)] [41]. Gallicchio et al. (2011) demonstrated that exposure to the mist of cleaning products had a significant association with the frequent reporting of sleep disturbances among cosmetologists [OR = 1.64 (95%CI: 1.10–2.45)] [34]. In the study of London et al. (1998), pesticide applicators (mist) compared with non-applicators reported significantly more sleepiness [39]. Chuang et al. (2018) observed that welding workers who were exposed to metal fumes were more time awake as compared to office workers (P < 0.05) [17]. Studies by Lee et al. (2020) indicated that severe exposure levels to dust elevated insomnia or sleep disturbance risk among the general working population [OR = 1.52 (95%CI: 1.25–1.85)] [37]. Mohammadyan et al. (2019) concluded a significant relationship between the quality of sleep, air lead contents (P = 0.02), and blood lead levels among soldering workers (P = 0.03) [40]. Two studies also did not report significant relationships [42,44]. Powell et al. (2021) in their studies revealed that the prevalence of obstructive sleep apnea (OSA) was high in both groups of exposed and non-exposed to smoke from burn pit, but it has no significant difference (P = 0.83) [42]. Qin et al. (2014) concluded that insomnia had no significant difference between welders exposed to fume and non-dust workers (P = 0.126) [44]. Fuhrimann et al. observed that there are positive associations between any pesticide applications in the last 7 days and all three MOS-SS indices [33]. Zheng et al. found that agricultural employees had a significantly higher risk of insomnia, especially among those who did not wear protective gloves [52].

Among 9 articles of moderate quality, eight studies (88.89 percent) showed a meaningful impact on sleep among workers of different occupations due to exposure to air aerosols. Among these studies, one analyzed the effect of smoke exposure on sleep, three studies investigated the effect of mist exposure on sleep, five studies examined the effect of fume exposure on sleep, and three studies examined the effect of dust presence on sleep. The negative consequences related to the state of sleep were sleep disturbances, sleepiness, obstructive sleep apnea, insomnia, and poor sleep quality. Pan et al. (2000) revealed in their studies that being exposed to non-industrial office dust might be responsible for sensory symptoms in the eyes and nose, and physiological changes [43]. The study performed by Jay et al. (2017) revealed that among general workers those with non-standard working hours were more exposed to dust, smoke, and fume in the workplace and as a consequence reported more frequently inadequate sleep and excessive sleepiness [36]. Sohn et al. (2001) concluded that pesticide poisoning due to exposure to mist of this substance was significantly related to sleeplessness among farmers (P < 0.01) [47]. The results of Sunderram et al. (2019) research revealed that new and worsening chronic rhinosinusitis due to exposure to toxic dust and fume, compared with no chronic rhinosinusitis, was a significant risk factor for obstructive sleep apnea in rescue and recovery workers [OR = 1.80 (95%CI: 1.18–2.73)] [48]. Li et al. (2019) observed that insufficient protective behavior in pesticide use was stated as the cause of decreased sleep quality and increased prevalence of nightmares among greenhouse farmers [38]. Eikinci et al. (2014) reported that insomnia was the most common systemic symptom among workers exposed to mercury fume in the battery industry [32]. In the study of Walczak et al. (2010), sleeplessness was dominant in nervous system functional disorders among workers who were chronically exposed to arsenic fume in copper smelting factories [51]. Jamal et al. (2016) concluded that the rates of insomnia were significantly higher in the pesticide (organophosphate) sprayers than controls among farmers (P = 0.03) [35]. Only one study also did not report a significant relationship. Bowler et al. (2007) observed that exposure to manganese fume showed no significant association with sleep disturbance among bridge welders [31].

Among three articles with low quality, two studies (66.67 percent) indicated that exposure to aerosols had a significant negative effect on sleep disturbances among workers in different occupations. Among these studies, two investigated the effect of mist exposure on sleep and one study researched the effect of dust on sleep. The sleep consequence was sleepiness. In the study of Tripathi et al., (1989) significant deleterious effects due to exposure with mist of solvents were noted on sleepiness among painters [49]. Sosan et al. (2009) also observed that some farmers complained of sleeplessness symptoms for medical treatment, which are suggestive of chronic pesticide poisoning due to exposure to insecticide mist [46]. Takam et al. (1988) concluded that there were no significant differences in sleep-related symptoms between the workers exposed to cotton dust and the control group in the textile industry [50].

**Fig 6:**
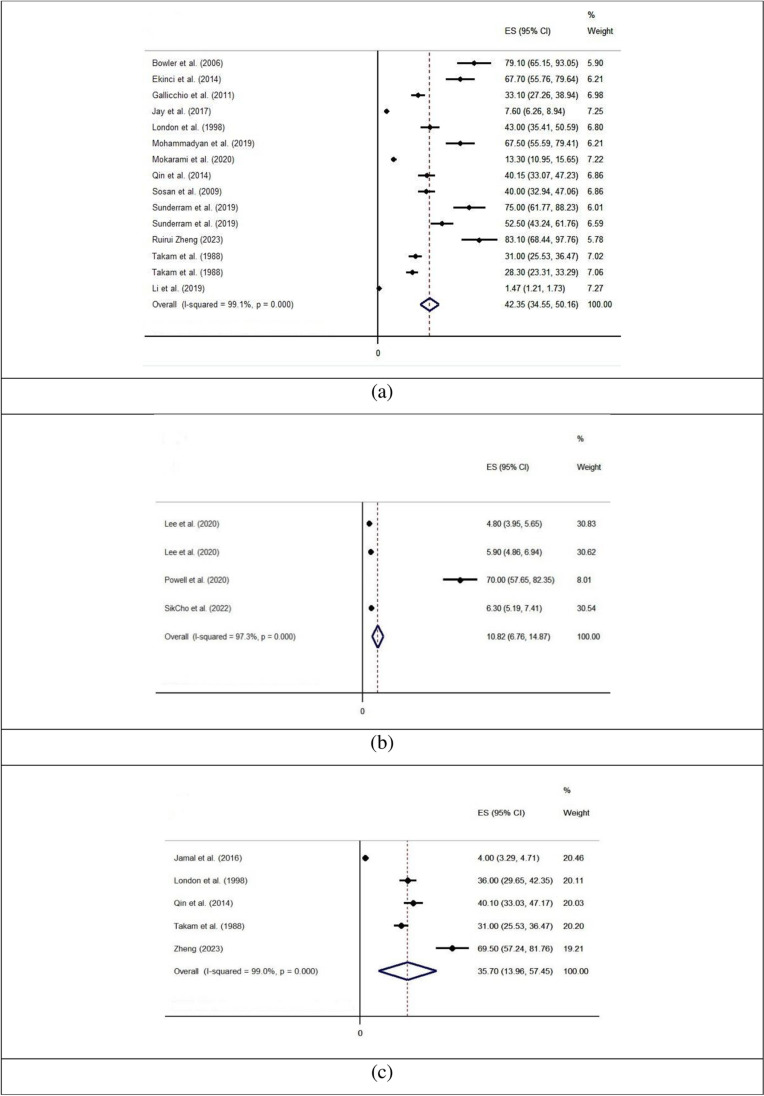
The pooled values of the prevalence of sleep problems due to occupational exposure to aerosols: (a) for cross-sectional studies, (b) for cohort studies, and (c) case control studies.

Fig 6 shows the pooled values of the prevalence of sleep problems due to exposure to aerosols. The results of the heterogeneity evaluation based on the value of I^2^ showed that there is a high heterogeneity for forest plots of cross-sectional studies, cohort studies, and case control studies. The results of the meta-analysis showed that the pooled values of the prevalence in cross-sectional studies, cohort studies, and case control studies were 42.35 (95% CI [34.55, 50.16]), 10.82 (95% CI [6.76, 14.87]), and 35.70 (95% CI [13.96, 57.45]), respectively. Moreover, Table 3 completely describes the results of the subgroup analysis for the prevalence. The pooled values of prevalence in cross-sectional, cohort, and case control studies were significantly greater in countries with high incomes compared to those with low and medium incomes, in North America, the Middle East, and Europe compared to other regions, and in after 2010 compared to 2010 or earlier. In terms of aerosol type, the pooled values of prevalence were the greatest values for fumes and smoke.

**Table 3:** The results of subgroup analysis for the prevalence of sleep problems due to occupational exposure to aerosols.

Subgroup analysis						
Cross-sectional studies	Subgroup	Category (number of studies)	Pooled prevalence (%)	*I*^2^ (%)	*Q* statistic	*p* of heterogeneity
			[95% CI]		(*df*)	
	Income level	High income (8)	47.81 [30.88, 64.75]	98.6	7	<0.0001
		LMICs (7)	38.17 [24.06, 52.27]	99.1	6	<0.0001
	Region	Europe (3)	45.66 [25.92, 65.41]	95.9	2	<0.0001
		East/southeast Asia/Oceania (3)	14.48 [6.64, 22.32]	99	2	<0.0001
		Middle East (3)	49.20 [6.08, 92.31]	98.6	2	<0.0001
		North America (4)	59.30 [37.21, 81.38]	95.1	3	<0.0001
		Africa (2)	41.39 [36.22, 46.55]	0	1	0.57
	Study date	In or before 2010 (5)	42.70 [31.41, 54.00]	92.5	4	<0.0001
		After 2010 (10)	41.25 [32.96, 49.54]	99.1	9	<0.0001
	Type of pollutant	Fume (4)	63.08 [44.07, 82.10]	91.9	3	<0.0001
		Mist (5)	39.58 [14.02, 65.14]	99.1	4	<0.0001
		Dust (6)	32.91 [21.80, 44.03]	98.3	5	<0.0001
		Smoke (-)	–	–	–	–
Cohort studies	Income level	High income (4)	10.81 [6.76, 14.86]	97.3	3	<0.0001
		LMICs (-)	–	–	–	–
	Region	Europe (-)	–	–	–	–
		East/southeast Asia/Oceania (3)	5.61 [4.68, 6.53]	61.4	2	0.075
		Middle East (-)	–	–	–	–
		North America (1)	70.00 [57.65, 82.34]	–	0	–
		Africa (-)	–	–	–	–
	Study date	In or before 2010 (-)	–	–	–	–
		After 2010 (4)	10.81 [6.76, 14.86]	97.3	3	<0.0001
	Type of pollutant	Fume (-)	–	–	–	–
		Mist (-)	–	–	–	–
		Dust (2)	5.30 [4.23, 6.38]	61.3	1	0.108
		Smoke (1)	37.84 [-24.58, 100.26]	99	1	<0.0001
Case control studies	Income level	High income (2)	49.84 [12.12, 87.56]	96.8	1	<0.0001
		LMICs (3)	26.54 [0.54, 53.63]	99	2	<0.0001
	Region	Europe (2)	49.84 [12.12, 87.56]	96.8	1	<0.0001
		East/southeast Asia/Oceania (2)	21.87 [13.50, 57.24]	99	1	<0.0001
		Middle East (-)	–	–	–	–
		North America (-)	–	–	–	–
		Africa (1)	36.00 [29.65, 42.35]	–	0	–
	Study date	In or before 2010 (2)	33.22 [28.35, 38.10]	26.9	1	0.242
		After 2010 (3)	37.46 [1.13, 73.78]	99	2	<0.0001
	Type of pollutant	Fume (1)	40.10 [33.02, 47.17]	–	0	–
		Mist (3)	36.01 [2.73, 69.28]	99	2	<0.0001
		Dust (1)	31.00 [25.53, 36.46]	–	0	–
		Smoke (-)	–	–	–	–

**Fig 7:**
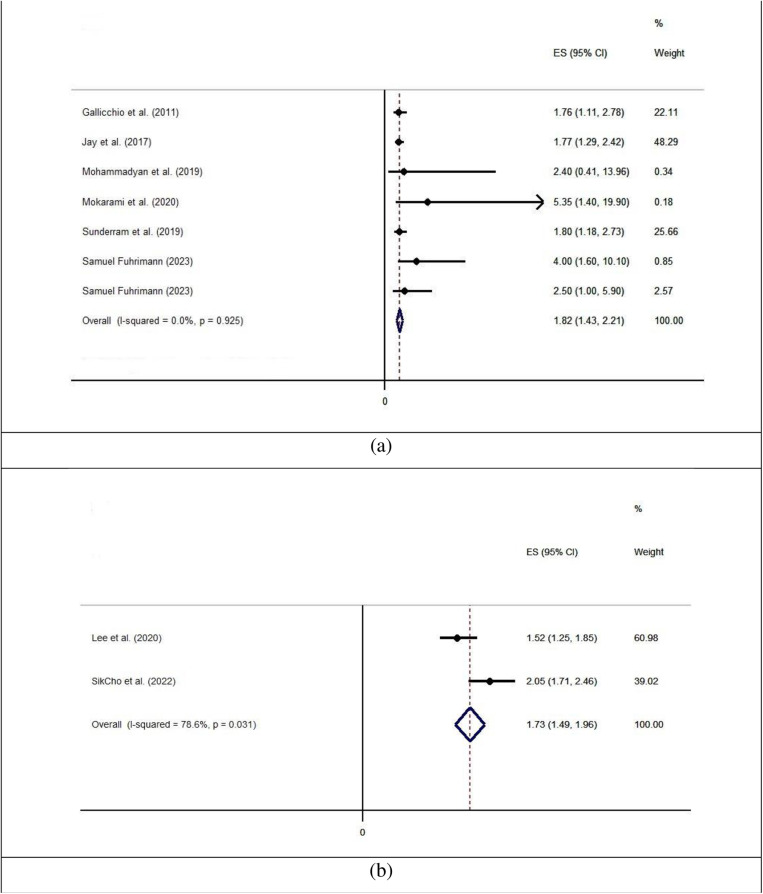
The pooled values of odds ratios related to sleep problems due to occupational exposure to aerosols: (a) for cross-sectional studies and (b) for cohort studies.

Fig 7 depicts the pooled values of odds ratios related to sleep problems due to exposure to aerosols. The results of the heterogeneity evaluation based on the value of I^2^ revealed that there are low and high heterogeneities for forest plots of cross-sectional studies and cohort studies, respectively. The results of the meta-analysis indicated that the pooled values of the odds ratio in the cross-sectional and cohort studies were 1.82 (95% CI [1.43, 2.21]) and 1.73 (95% CI [1.49, 1.96]), respectively. Moreover, Table 4 completely reports the results of the subgroup analysis for the odd ratios. The pooled values of odds ratios in cross-sectional studies and cohort studies were significantly higher in countries with low and medium incomes compared to those with high incomes, in the Middle East compared to other regions, and after 2010 compared to after this time. In terms of aerosol type, the pooled values of odds ratios were the greatest values for fume and smoke.

**Table 4:** The results of subgroup analysis for the odd ratios related to sleep problems due to occupational exposure to aerosols.

Subgroup analysis						
Cross-sectional studies	Subgroup	Category (number of studies)	Pooled prevalence (%)	*I*^2^ (%)	*Q* statistic	*p* of heterogeneity
			[95% CI]		(*df*)	
	Income level	High income (3)	1.77 [1.37, 2.17]	15.4	2	0.99
		LMICs (4)	2.94 [0.96, 4.92]	0	3	0.88
	Region	Europe (-)	–	–	–	–
		East/southeast Asia/Oceania (1)	1.77 [1.20, 2.33]	–	0	–
		Middle East (2)	3.43 [-2.03, 8.89]	0	1	0.61
		North America (2)	1.78 [1.21, 2.35]	0	1	0.94
		Africa (2)	2.87 [0.75, 4.99]	0	1	0.54
	Study date	In or before 2010 (1)	1.76 [0.92, 2.59]	–	0	–
		After 2010 (6)	1.83 [1.39, 2.28]	0	5	0.86
	Type of pollutant	Fume (1)	2.40 [-4.37, 9.17]	–	0	–
		Mist (3)	1.90 [1.13, 2.68]	0	2	0.52
		Dust (3)	1.78 [1.33, 2.24]	0	2	0.75
		Smoke (-)	–	–	–	–
Cohort studies	Income level	High income (2)	1.72 [1.49, 1.96]	78.6	1	0.03
		LMICs (-)	–	–	–	–
	Region	Europe (-)	–	–	–	–
		East/southeast Asia/Oceania (2)	1.72 [1.49, 1.96]	78.6	1	0.03
		Middle East (-)	–	–	–	–
		North America (-)	–	–	–	–
		Africa (-)	–	–	–	–
	Study date	In or before 2010 (-)	–	–	–	–
		After 2010 (2)	1.72 [1.49, 1.96]	78.6	1	0.03
	Type of pollutant	Fume (-)	–	–	–	–
		Mist (-)	–	–	–	–
		Dust (1)	1.52 [1.22, 1.82]	–	0	–
		Smoke (1)	2.05 [1.67, 2.42]	–	0	–

### Assessment *of* risk *of* bias *in the* studies

**Fig 8:**
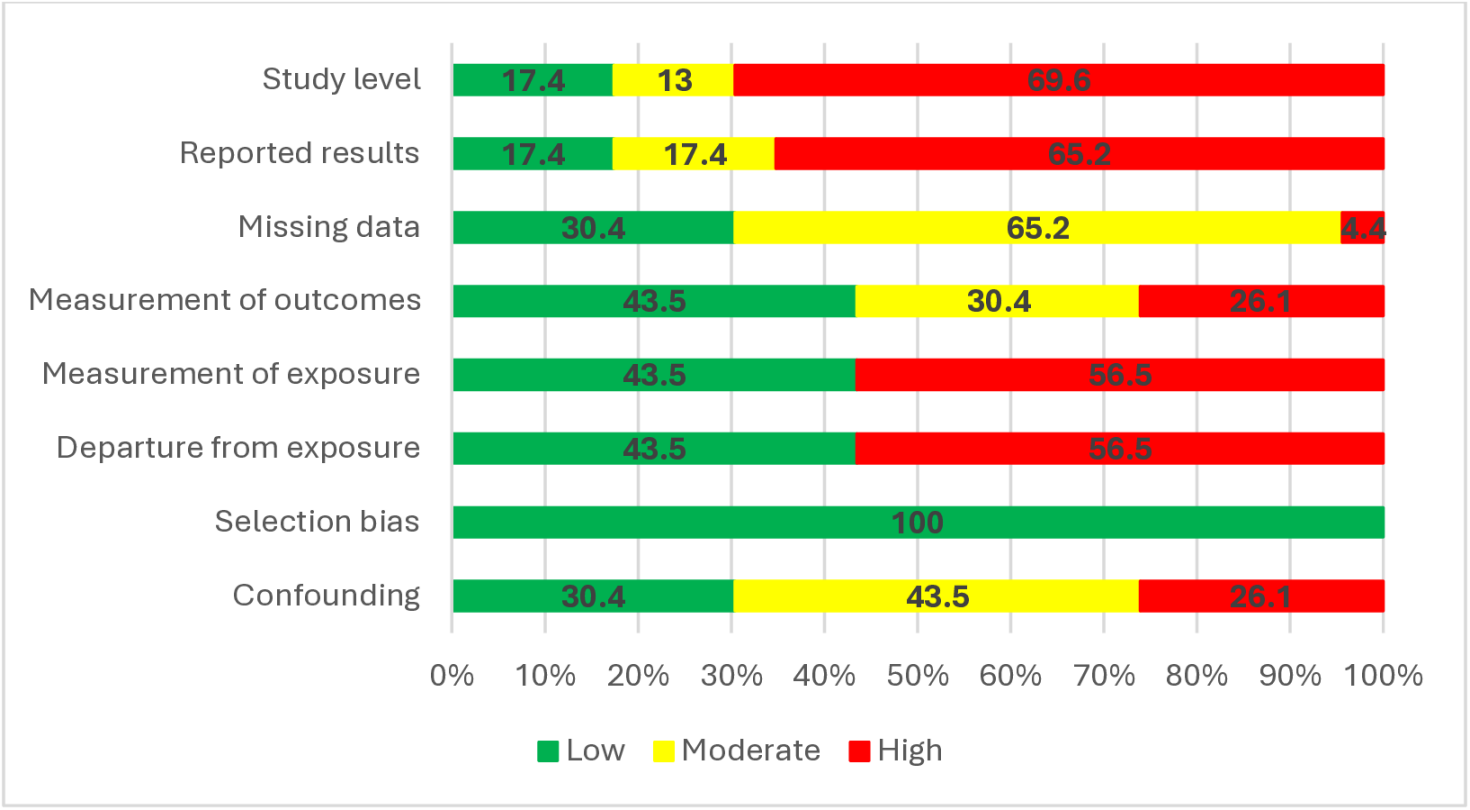
Assessment of risk of bias in the studied articles related to sleep problems due to occupational exposure to aerosols.

The evaluation results of the seven ROB criteria for each of the articles have been reported in Table 5. The general status of the risk of bias in the studied articles is summarized in Fig 8. Out of the 23 selected articles, 69.6% (N = 16) were given a high ROB rating, 13.0% (N = 3) were rated as moderate, and 17.4% (N = 4) were rated as low ROB. The highest percentage of high ROB rating was observed in the criteria of “reported results” with 65.2% (N = 15). Also, “selection bias” was observed at a low level in 100% of the studies.

**Table 5:** Evaluation of RoB in the reviewed studies.

First Author	Confounding	Selection bias	Departure from exposure	Measurement of exposure	Measurement of outcomes	Missing data	Reported results	Study level
Bowler et al. (2006)	High	Low	Low	Low	High	Moderate	High	High
Chuang et al. (2017)	Low	Low	Low	Low	Low	Low	Low	Low
Ekinci et al. (2014)	High	Low	Low	Low	High	Moderate	Moderate	High
Gallicchio et al. (2011)	High	Low	High	High	High	Moderate	High	High
Jamal et al. (2016)	High	Low	High	High	High	Moderate	High	High
Jay et al. (2017)	High	Low	High	High	Low	High	High	High
Lee et al. (2020)	High	Low	High	High	High	Moderate	High	High
Li et al. (2019)	Moderate	Low	High	High	Moderate	Moderate	High	High
London et al. (1998)	Moderate	Low	High	High	Moderate	Low	High	High
Mohammadyan et al. (2019)	Low	Low	Low	Low	Low	Low	Low	Low
Mokarami et al. (2020)	Low	Low	Low	Low	Low	Low	Low	Low
Powell et al. (2020)	Low	Low	High	High	Moderate	Moderate	High	High
Pan et al. (2000)	Low	Low	Low	Low	Moderate	Low	Moderate	Moderate
Qin et al. (2014)	Moderate	Low	High	High	Moderate	Moderate	High	High
SikCho et al. (2022)	Moderate	Low	High	High	Moderate	Moderate	High	High
Sosan et al. (2009)	Moderate	Low	High	High	Low	Moderate	High	High
Sohn et al. (2001)	Moderate	Low	High	High	Moderate	Moderate	High	High
Sunderram et al. (2019)	Moderate	Low	High	High	Low	Moderate	High	High
Tripathi et al. (1989)	Low	Low	Low	Low	Low	Moderate	Moderate	Moderate
Takam et al. (1988)	Moderate	Low	Low	Low	Low	Moderate	Moderate	Moderate
Walczak et al. (2010)	Moderate	Low	Low	Low	High	Moderate	High	High
Samuel Fuhrimann (2023)	Low	Low	Low	Low	Low	Low	Low	Low
Ruirui Zheng (2023)	Moderate	Low	High	High	Low	Low	High	High

## Discussion

Until now, several studies have examined the impact of occupational exposure to aerosols on human sleep. This research, however, was performed for various exposure conditions and occupation characteristics that resulted in obtained findings. Therefore, the presented study aimed to conduct a systematic review and meta-analysis on the correlation between occupational exposure to aerosols and human sleep. Based on the results, 23 articles were entered into our study. The included research was carried out in different occupations in 15 countries.

In this study, eleven articles were classified as high quality, nine studies as moderate quality, and three studies as low-quality studies. Therefore, the study quality appears to influence the findings, although a small number of low-quality studies were involved. However, the results of the bias assessment indicated that 69.6% of the research had a high risk of bias (ROB), while 13.0% had a moderate ROB, and only 17.4% were considered to have a low ROB. The discrepancy between the results obtained by the two instruments may be because the criteria assessed in ROBINS-E and JBI instruments are somewhat different. Also, there is a difference in classifications of quality and bias risk levels in these instruments. So the classification of bias in the ROBINS-E tool is stricter than in the JBI checklist. In the ROBINS-E tool, even if any single criterion received a “high” rating, an overall classification of “high” risk of bias was assigned. The low frequency of studies with a low ROB may be attributed to several factors including the reliance on self-reported data or questionnaires for gathering information [53–55], the omission of details regarding pollutant levels, and the prevalence of sleep disturbances [56–58], failure to report exposure status [56,59], utilization of similar methodologies [60, 61], and instances where results were imperfectly provided [62–64]. Furthermore, the investigation conducted by Han et al. revealed considerable concerns about exposure assessment in the rationale/notes subsection, as well as a high ROB in the subsection concerning the selection process of study participants [65].

In our systematic review and meta-analysis, 9 out of 11 studies of high quality (81.82 percent), 8 out of 9 articles of moderate quality (88.89 percent), and 2 out of 3 studies of low quality (66.67 percent) indicated that occupational exposure to aerosols substantially affected the sleep among workers in different occupations. In these studies, the effects of occupational aerosol exposure on sleep were also introduced as sleep disturbance, sleepiness, poor sleep quality, insomnia, and obstructive sleep apnea. Therefore, occupational aerosol can affect the sleep of the workers. The highest sleep problems due to this exposure were also seen because of exposure to pesticide mist, manganese fume, and toxic dust and fumes, respectively. Postuma et al. also found that pesticides can cause sleep behavior disorders with an odds ratio of 2.23 [66]. It can make this effect through various pathways that the present study has identified. Occupational aerosol exposure can affect night sleep through four main paths, including the neurobehavioral effect due to chemical aerosol exposure on nighttime sleep [67], the psychological effects due to aerosol exposure on nighttime sleep [15], the effect of chronic illnesses due to aerosol exposure on nighttime sleep [16], and the effect of serotonin imbalance due to aerosol exposure on the nighttime sleep [68]. It should be noted that in this regard, the type, shape, and size of the aerosol determines the rate of its entry into the central nervous system and the chemical composition of the aerosol specifies the type and rate of effect [69].

Among mechanisms explaining the effect of daytime occupational aerosol exposure on sleep, the neurobehavioral effect due to chemical aerosol exposure is considered the most possible. This mechanism seems to have specific importance for all aerosols [67]. The most common fumes in the workplace include manganese, iron, zinc, nickel, chromium, lead, cadmium, cobalt, and magnesium [70]. Exposure to some of these fumes such as iron, lead, and particularly manganese, can increase the risk of neurobehavioral disorders among welders [71]. Manganese (Mn) is known to be neurotoxic for the central nervous system [72]. Bowler et al. (2007) revealed that exposure to manganese fume in some welders was associated with neuroglial consequences, like sleep disturbances [72]. Studies by Walczak et al. (2010) indicated that sleeplessness is a prevailing symptom of nervous system functional disorders during chronic exposure to arsenic fume by workers in copper smelting factories [51]. On smoke, it consists of ultrafine particles that can enter the central nervous system and cause neuroinflammation, thereby leading to the consequences of sleep disorders [73]. Ehsanifar et al. concluded that exposure to nanoscale diesel exhaust particles can be associated with neuroinflammation [73]. In addition, smoke is often accompanied by carbon monoxide and carbon dioxide, which can cause neurotoxicity. Rutchik and Ratner concluded that persons exposed to monoxide carbon experience neurological symptoms [74]. Exposure to mist of some chemical substances can also influence the nervous system directly or indirectly and is associated with neurobehavioral effects, such as sleep problems [14]. These effects depend on chemical substances in aerosol. The various studies investigated the different aerosols from a variety of chemical substances. The results of an occupational study revealed that some organic compounds used in cosmetic salons might induce headaches, fatigue, sleep disturbances, and difficulties with maintaining concentration [75]. Indeed, the study performed by Yu et al. (2004) indicated that occupational exposure of printing workers to organic solvent mixtures increased the risk of inducing neurological symptoms [76]. Viaene et al. (2009) concluded that occupational exposure to solvents, such as aerosol or vapors, can cause sleep disturbances [75]. Tripathi et al. (1989) also reported neurobehavioral disturbances, such as sleepiness, in the workers in high-pressure spray painting [49]. The study performed by London et al. (1998) indicated that long-term exposures to organophosphate pesticides can be associated with neurological symptoms, such as sleepiness, in farm workers [39]. Li et al. (2019) reported that there are association between the use of pesticide sprays and neurologic symptoms, which can cause consequences such as sleep disorders [38]. The results of other studies revealed that the frequency of pesticide usage was positively correlated with the occurrence of neurobehavioral and neurologic symptoms [77, 78]. Indeed, different substances can be entered into the body through inhalation during occupational operations and cause alterations in the central nervous system functioning [79]. On simple dust also, it seems that this mechanism has not importance but for some ultrafine dust, such as manganese and copper dust can be associated with neurobehavioral symptoms [80].

As a second pathway, exposure to aerosol can also be associated with psychological effects, including annoyance, health concerns, and anxiety because of direct exposure to dangerous aerosol in poor working conditions [15]. This mechanism can be created by various types of aerosols, particularly aerosols with acute effects [81]. The health concerns or anxiety due to aerosol exposure can cause stressful conditions, increase stress reactions, and activate the hypothalamic-pituitary-adrenal axis and rumination [82]. Rhythmic adrenal cortisol can be disrupted due to increasing adrenal secretory activity caused for instance by stress [83]. Similar to the known mechanism of the effect of noise annoyance on psychological problems by causing sustained central autonomic arousal as well as disruption of the dopamine pathway [84], exposure to occupational aerosols may also affect negatively mental health via triggering sustained nervous stimulation, which is related to cortical activation. The negative impact of exposure to aerosols on mental health is called “aerosol annoyance”. This concept in mental health can be described by a neurocognitive mechanism. The predisposing factors, like stress factors in workplaces, associated with somatic, cognitive, and cortical activation, are interrelated to perpetuating factors, for instance, extension of sleep time due to obstacles to de-arousal from cortical arousal [85]. These changes in the human body can impair sleep and sleepiness. The results of the previous research revealed that chronic stress can be responsible for severe health consequences, such as sleep problems [86, 87]. In their study, Lee et al. (2020) revealed the relationship between occupational exposure to dust among Korean workers and mental health symptoms occurrence, like insomnia or sleep disturbance [37].

As the third pathway, exposure to the aerosols can result in chronic illnesses, such as local inflammation of airways, dryness of the throat, coughing, wheezing, tightness in the chest, difficulty in breathing up to chronic bronchitis, which disturbs the person’s comfort and leads to sleep disorders [16]. This mechanism also has importance for all types of aerosols, particularly allergenic aerosols. Long-term exposure to aerosols might result in sleep disturbances, like sleep apnea syndromes [75]. As a suggestive mechanism, environmental exposures to dust and other aerosols could provoke OSA through inflammatory changes of the upper inhalational track [88]. Moreover, an increase in apnea index during sleep in persons who experienced seasonal allergic rhinitis during the pollen season [89] was reported. Other papers showed clinically significant obstructive sleep apnea caused by occupational sensitization to guar gum dust [90]. Symptoms of allergic and non-allergic rhinitis are a risk factor for OSA [91]. Among potential mechanisms an increased risk of OSA related to CRS and the effect of nasal obstruction on collapsibility of the nasopharynx downstream is mentioned. An increase in nasal resistance is reported to be the most likely mechanism and inflammation related to an elevated nasal resistance is claimed to be the cause [48]. The impact of inhalational exposure to various aerosols in workplaces was indicated as the cause of obstructive sleep apnea (OSA) reported in previous studies. Meta-analysis performed by Schwartz et al. (2015) indicated the impact of exposure to solvents on increased risk of OSA occurrence [92]. Mølhave et al. (2000) indicated a significant impact of dust, in questionnaire evaluations, as well as indicated that the reported state of discomfort increased with increasing dust exposure. This study also indicated negative effects on the eyes and nose caused by dust exposure: epithelium damage, tear film stability, foam formation in canthus, and nasal volume [43]. Other respiratory disturbances reported in the studies as the exposure to metal fumes consequences were as follows: pulmonary function, throat dryness, chest tightness, coughing, wheezing, difficulty in breathing, and chronic bronchitis [93]. The results of a study conducted by Sunderram et al. (2019) indicated that chronic rhinosinusitis (CRS) due to exposure to significant amounts of dust was a significant risk factor for obstructive sleep apnea in rescue and recovery workers [48]. Other studies revealed the adverse effects of heavy metals exposure, including lead, on the quality of sleep in the form of physical illnesses such as digestive disorders, anemia, and neurological diseases [94]. Mohamadyan et al. (2019) concluded that there was a significant relationship between exposure to lead with digestive disorders and sleep quality [40]. Also, Sosan et al. (2009) suggested that the symptoms of illness observed among farmers in their study, like difficulties in sleeping after spraying operations, could be caused by insecticide poisoning as the result of occupational exposure during the usage of these chemicals [46].

As the fourth pathway, exposure to some aerosols can cause serotonin imbalance. Being one of the most important chemicals that are responsible for sleep and awake cycle regulation in the brain, lowering its levels are to resulting in sleepiness and sleep disturbances [68]. The results of a study performed by Chuang et al. (2018) indicated that personal exposure to fumes including Cd, Co, Cu, Mn, Ni, and Pb in welding workers was associated with a decrease in their urinary serotonin and an increase in their awake time during sleep [17]. Also, chronic exposure to dust can lead to chronic inflammation [95] that can disrupt hormonal regulation in this way [96]. Recent studies also indicated the negative impact of heavy metals like Mn, Pb, and Cd on sleep and the circadian cycle [17,97]. Other studies indicated that exposure to heavy metals, especially Pb, could directly disturb the sleep cycle through for instance disordering of secretion of sleep hormones, such as serotonin and cortisol [17,94]. The cohort study performed by Braun et al. (2014) revealed a significant correlation between blood lead levels among pregnant women and cortisol secretion [98]. McIntosh et al., (1988) indicated that exposure to lead to be the main cause of secretion of catecholamine, dopamine, epinephrine, and epinephrine in rats [99].

The results of the meta-analysis also showed that the pooled values of the odds ratio related to sleep problems due to exposure to aerosols for cross-sectional studies and cohort studies were 1.82 and 1.73, respectively. About other harmful agents in the workplace, Heo et al. concluded that physical risk factors could cause sleep disturbance with an odds ratio of 1.47. In this study, psychosocial agents could affect sleep quality of workers with an odds ratio between 1.45 and 2.93 [15]. The results of a study performed by Virtanen et al. showed that long working hours can be associated with an odds ratio of 3.24 for shortened sleep and 2.23 for early morning awakenings [100]. Park et al. found that exposure to physical agents, including vibration, noise, and high and low temperature, could cause sleep disturbance with an odds ratio between 1.69 and 3.52 [101]. Heiskel et al. observed that exposure to solvents can lead to obstructive sleep apnea with an odds ratio of 1.2 [102]. These findings indicate that chemical agents like other occupational risk factors can be significantly associated with sleep problems.

The results of the meta-analysis showed that the pooled values of the prevalence of sleep problems due to exposure to aerosols for cross-sectional, cohort, and case control studies were 42.35, 10.82, and 35.70 percent, respectively. Previous studies revealed that the prevalence of sleep problems in the working population was in the range of approximately 18 percent in Europe to 23 percent in the United States [103,104]. Cho et al. observed that the prevalence of sleep disturbance due to exposure to physical or chemical factors was between 10 and 26 percent [45]. Bertrais et al. concluded that exposure to occupational risk factors was associated with a higher prevalence of sleep problems by nearly 30 percent in men [105]. The prevalence of sleep problems among workers in the present study was approximately near to those in other studies.

The results of subgroup analysis showed that the pooled values of prevalence and odds ratios were significantly higher in countries with low and medium incomes, North America and the Middle East, and in 2010 or earlier. In countries with low and middle incomes, old industrial machinery with suboptimal energy efficiency is used, which can produce higher concentrations of pollutants. Moreover, some of these nations rely on energy sources such as coal and mazut, which are less efficient and play a role in health complications due to air pollution. Furthermore, the insufficient implementation of policies and regulations to prevent emissions of pollutants, because of the exorbitant expenses for pollution control, exacerbates the risk of exposure to pollutants [106]. Nevertheless, it is important to recognize that developed countries also have some reasons for high pollutants, such as more extensive industrial activities in developed nations [107]. These reasons might partially explain the outcomes of the present studies. Also, the results showed that the values of odds ratios and prevalence were the highest values for fumes and the lowest values for dust. As mentioned, it seems that the chemical composition of aerosols has higher importance than types of aerosols [69]. However, the type of aerosols can affect the absorption and entrance of substances to various body regions [108]. Fumes can create sleep problems through all stated four mechanisms because of ultrafine size, shape, chemical composition, and prevalent emission [108]. The low values for dust may be due to the fact that most dusts are coarse in size and simple in chemical composition, which cannot cause very serious disturbances. However, it should be noted that some dusts of dangerous chemicals can cause serious consequences [69].

In examining the relationship between exposures to different types of aerosols and sleep problems, there are several confounders that should be mentioned, and their role must be investigated in future studies. One group of confounders is exposure to other pollutants affecting sleep quality, such as gases and vapors [75]. Another group is the use of drugs, alcohol, smoking, mental disorders, stress, heat, cold, noise, waves, and other occupational and lifestyle factors that can influence sleep quality in humans [109,110].

## Limitations

Our work also has some limitations. Of obtained 23 research only three studies were cohort studies. The prospective studies allowed for higher confidence in examining exposure values to aerosols as compared to retrospective studies, to which the recall fallacy issue was related. Also, the specific chemical composition of aerosols was not evaluated in the reviewed studies. Depending on the exposure, the toxicity of aerosols can be very high, both in general terms and specifically in relation to sleep disorders. Thus, we can imagine that by analyzing only the 4 types of particles were evaluated in the present study, without analyzing the chemical composition. The impact of the effect of confounding factors, like other harmful agents in the workplace, was not investigated in some analyzed studies that could influence the impact of aerosol exposure. As another limitation, the tools used in the reviewed studies for the assessment of sleep quality were different. In addition, the lack of searches in reference lists, gray literature, and the inclusion of other languages may cause publication bias.

## Conclusion

Analysis performed in this systematic review and meta-analysis based on 17 (80.95%) out of 23 studies indicated that exposure to occupational aerosols can significantly affect the sleep of employees from various occupations. Occupational exposure can lead to a variety of sleeping problems. Four pathways of these sleeping disturbances were described, including the neurobehavioral effect due to chemical aerosol exposure on the time sleep during the night, the psychological effects due to aerosol exposure on the nighttime sleep, the effect of chronic illnesses due to aerosol exposure on the time sleep during the night, and the effect of serotonin imbalance due to aerosol exposure on the time sleep during the night. The results of this study can be useful in preventing sleep disorders related to occupational environments.

## Abbreviations

PRISMA: Preferred reporting items for systematic reviews and meta-analyses
ROBINS-E: The Risk of Bias in Non-randomized Studies - of Exposure
JBI: Joanna Briggs Institute
OR: Odds ratio
MISS: Minimal insomnia symptom scale
ESS: Epworth sleepiness scale
OSA: Obstructive sleep apnea
CRS: Chronic rhinosinusitis
